# Psychological experiences of nurses caring for patients with COVID‐19: Integrative review based on qualitative research

**DOI:** 10.1002/nop2.1813

**Published:** 2023-05-19

**Authors:** Serah Lim, Hyeran Park, Sanghee Kim

**Affiliations:** ^1^ College of Nursing Yonsei University Seoul Korea; ^2^ College of Nursing and Mo‐Im Kim Nursing Research Institute Yonsei University Seoul Korea

**Keywords:** COVID‐19, integrative review, nurses, psychological experiences, qualitative research

## Abstract

**Aim:**

To analyse the literature on nurses' psychological experiences caring for COVID‐19 patients, focusing on qualitative research.

**Design:**

An integrative review.

**Review Methods:**

Whittemore & Knafl's approach was used.

**Data Sources:**

Six databases were searched using the terms ‘nurses’, ‘psychological experiences’ and ‘COVID‐19’.

**Results:**

Ten studies were selected and analysed. Five characteristics related to nurses' negative psychological experiences, four characteristics related to positive psychological experiences and seven coping strategies of nurses were identified.

**Conclusion:**

This study demonstrated the need for psychological, social, financial and organizational support for nurses to improve mental well‐being and the level of nursing care.

No Patient or Public Contribution.

## 1 INTRODUCTION

Severe acute respiratory syndrome coronavirus‐2 (SARS‐CoV‐2) first appeared in December 2019, rapidly spreading worldwide, and the World Health Organization declared coronavirus disease 2019 (COVID‐19) a pandemic on 11 March 2020 (WHO, 2020). Because its transmission routes and disease progression were inadequately known and appropriate treatments were still in development, there was uncertainty and fear in all areas of life (Muz & Erdoğan Yüce, 2021). In the pandemic crisis, countries worldwide have implemented measures such as quarantine, social distancing and school closures, completely changing people's daily lives (Shanafelt et al., 2020). Healthcare workers directly involved in screening, treatment and care are the most affected by COVID‐19 (Lai et al., 2020; Sun et al., 2020); they face the threat of disease contagion, overwhelming workloads and psychological distress (Firew et al., 2020; Lai et al., 2020).

Front‐line nurses play an essential role in caring for and have the most contact with COVID‐19 patients (Galehdar et al., 2020) and are at risk of developing psychological concerns (Firew et al., 2020; Hu et al., 2020; Lai et al., 2020). Previous studies showed that nurses experience higher levels of stress, anxiety, depression, post‐traumatic stress disorder (PTSD) and sleep disturbances than other healthcare workers during the pandemic (Shechter et al., 2020; Tam et al., 2004; Tang et al., 2017). Nurses experience anxiety about becoming infected while caring for patients and fear of spreading the virus to family members (Fernandez et al., 2020; Hu et al., 2020). Furthermore, its high morbidity and potential lethality may increase the perception of danger and anxiety (Lai et al., 2020). They also experience emotional exhaustion and stress from long working hours and heavy workloads caring for COVID‐19 patients (Leng et al., 2021; Mo et al., 2020). The diversity of the patients' care needs and the limited number of nurses increase the workload (Galehdar et al., 2020). They feel stressed when there are insufficient personal protective equipments (PPE) and infection control strategies, which increases their risk of exposure to the disease (Arnetz et al., 2020; Firew et al., 2020). Therefore, nurses experience multiple psychological pressures on the front line of the COVID‐19 pandemic.

## 2 BACKGROUND

Nurses are essential for the healthcare systems' pandemic response. They provide patient care, public health education, quarantine management, triaging and more (Seale et al., 2009). Although their roles are crucial during threatening events, not all nurses are prepared to work with highly contagious patients (Wong et al., 2010). Previous studies have shown that when nurses experience psychological stress and the fear of being infected, they are reluctant to work and may contemplate resignation (Martin, 2011; Seale et al., 2009; Wong et al., 2010). A recent study showed that nurses were nearly four times as likely to consider resigning during COVID‐19 as other healthcare workers (Chu et al., 2021). During the pandemic, an increased lack of nursing staff may lead to a severe shortage, becoming a possible threat to hospitals and emergency response infrastructure (Chu et al., 2021; Seale et al., 2009). Therefore, understanding nurses' psychological experiences during COVID‐19 and providing supportive resources is essential for high‐quality healthcare and effective institutional responses (Fernandez et al., 2020).

Although quantitative studies investigating healthcare workers' psychological experiences during COVID‐19 have reported increased depression and psychological distress (Arnetz et al., 2020; Leng et al., 2021; Mo et al., 2020; Shechter et al., 2020), there is a limit to an in‐depth understanding with only fragmentary variables. Additionally, studies specifically targeting nurses' psychological experiences during the pandemic are insufficient (Fernandez et al., 2020; Sun et al., 2020). Qualitative research about nurses with the closest contact with COVID‐19 patients is necessary to provide a detailed understanding of their struggles, which can provide fundamental data for developing supportive interventions.

### 2.1 Aim

This integrative review aimed to analyse nurses' psychological experiences during the COVID‐19 pandemic. The following research question was investigated: What are the psychological experiences of nurses caring for COVID‐19 patients? What are the coping strategies for the psychological experiences of nurses caring for COVID‐19 patients?

## 3 DATA SOURCES

### 3.1 Study design

This study was an integrative literature review synthesizing and analysing qualitative studies exploring nurses' psychological experiences caring for COVID‐19 patients.

### 3.2 Study procedure

Following the integrative review method suggested by Whittemore and Knafl (2005), five steps were implemented: problem identification, literature search, data evaluation, data analysis and presentation. The databases for the literature search were PubMed, Embase, CINAHL, Research Information Sharing Service (RISS), Korean Studies Information Service System (KISS) and the Korean Medical Database (KMBASE). The search terms were (1) ‘coronavirus (COVID‐19)’, (2) ‘nurses’ or ‘nurse practitioner’, (3) ‘psychological experience’ or ‘psychosocial experience’ and (4) ‘qualitative research’. The scope was set as December 2019–September 2021. The inclusion criteria were qualitative research related to the psychological experiences of nurses caring for COVID‐19 patients and published in English or Korean. Studies were excluded if the subjects included other healthcare workers or a specific group, such as nursing managers or male nurses, rather than general nurses. Studies focusing on nursing tasks or the nursing policy rather than nurses' psychological experiences were also excluded. Finally, unpublished manuscripts, quantitative studies, opinion‐based articles and letters to the editor were excluded.

**FIGURE 1:**
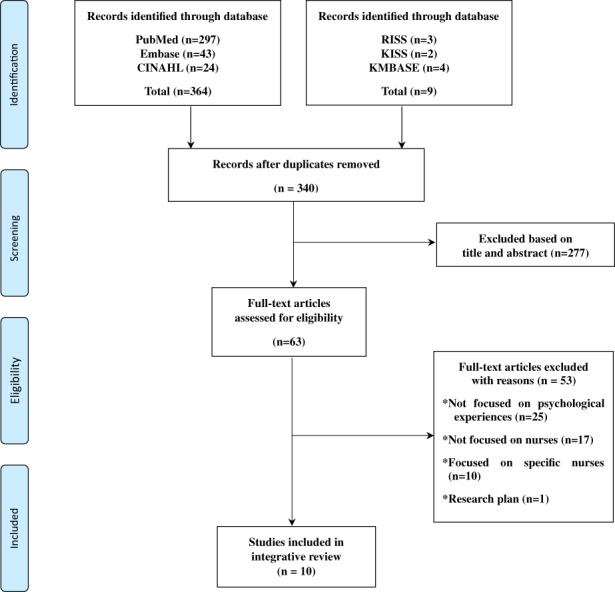
PRISMA flow diagram of study selection.

The literature search and selection process were presented using the Preferred Reporting Items for Systematic Reviews and Meta‐Analyses (PRISMA; Figure 1). A total of 373 articles were searched: PubMed‐297, Embase‐43, CINAHL‐24, RISS‐3, KISS‐2 and KMBASE‐4. They were registered in the Endnote program, and 33 duplicate articles were removed. Of the 340 articles, 330 articles that did not meet the inclusion criteria were excluded, and 10 articles were finally selected.

### 3.3 Quality appraisal

Two researchers independently evaluated the quality of the selected literature using the Critical Appraisal Skills Programme qualitative study checklist (http://www.casp‐uk.net/checklists). When there was a disagreement between the two researchers, a consensus was reached by discussing it with a third researcher. Appendix S1 presents the quality appraisals.

## 4 OVERVIEW OF THE ISSUES

## 5 RESULTS

This integrative review analysed the literature on nurses' psychological experiences caring for COVID‐19 patients, focusing on qualitative research using Whittemore and Knafl's approach. We identified five characteristics of nurses' negative psychological experiences, four characteristics of positive psychological experiences and seven coping strategies. These characteristics and participants' statements are described in detail below.

### 5.1 Characteristics of the studies

Table 1 lists the characteristics of the 10 studies. The studies were conducted in China (*n* = 5), Turkey (*n* = 2), Iran (*n* = 1), the USA (*n* = 1) and Canada (*n* = 1). A total of 163 nurses in Intensive Care Units, emergency rooms, designated wards and isolation units were included in the review data. Five studies used phenomenological methods: two were qualitative descriptive studies, one was a narrative and one was not mentioned.

**TABLE 1:** Characteristics of studies related to psychological experiences of nurses in COVID‐19 (*N* = 10).

No.	Author(year), country	Design	Sample description	Data collection	Reference of rigour
#1	Galehdar et al. (2020), Iran	No description, Content analysis	Nurses in ICU, CCU, Emergency, General ward and Infectious ward (*n* = 20)	Semi‐structured in‐depth telephone interviews	Lincoln and Guba (1985)
#2	Gordon et al. (2021), USA	Qualitative descriptive study Content analysis	Nurses in ICU (*n* = 11)	Semi‐structured face‐to‐face interviews	No description
#3	He et al. (2021), China	Phenomenology, Content analysis	Nurses who countermarched to the outbreak city for medical support (*n* = 10)	Semi‐structured in‐depth network visual interviews	No description
#4	Kackin et al. (2021), Turkey	Phenomenology	Nurses in ICU, Pulmonology, Haemodialysis centre, COVID‐19 clinic, Infectious ward and The Turkish Red Crescent (*n* = 10)	Semi‐structured online interviews	Lincoln and Guba (1985)
#5	Lapum et al. (2021), Canada	Narrative	Nurses in acute care hospital environments (*n* = 20)	Semi‐structured online interviews	No description
#6	Liu et al. (2020), China	No description	Nurses in ICU, General ward and Infectious ward (*n* = 15)	Semi‐structured in‐depth face‐to‐face interviews	Guba and Lincoln (1982)
#7	Muz et al. (2021), Turkey	Phenomenology	Nurses in pandemic ICU and pandemic ward (*n* = 19)	Semi‐structured video or audio interviews	Speziale et al. (2011)
#8	Sun et al. (2020), China	Phenomenology	Nurses in negative pressure ward (*n* = 20)	Semi‐structured face‐to‐face or telephone interviews	No description
#9	Zhang et al. (2020), China	Qualitative descriptive study	Nurses in isolation unit (*n* = 23)	Semi‐structured interviews	No description
#10	Zhang et al. (2021), China	Phenomenology	Nurses in COVID‐19 rescue task (*n* = 15)	Semi‐structured face‐to‐face interviews	Tong et al. (2007)

### 5.2 Negative psychological experiences of nurses caring for COVID‐19 patients

The studies revealed the various psychological experiences of nurses during the COVID‐19 pandemic. Table 2 describes negative experiences, including (1) anxiety about infection and fear of transmitting the virus to their families, (2) going into the fire, (3) feeling guilty, (4) the burden of overloaded and unfamiliar work and (5) exhaustion from wearing PPE.

**TABLE 2:** Negative psychological experiences of nurses in COVID‐19 (*N* = 10).

Themes	Quotations
Anxiety about infection and fear of transmitting the virus to their families	‘…This is a situation that I have to take care of people who may infect me at any moment, and then I may transmit the infection to my family, or the fact that I can even die…these thoughts are coming to me…what will happen to my child, to my life…’ (#1)
	‘But with these patients, because of the risk to myself (crying) and the risk of bringing home something to my family, it is very high stress’. (#2)
	‘I'm worried that I've been infected and whether I've infected my family because I went home last week (sighed deeply)’. (#6)
	‘I was worried at the beginning of course. Although I was wearing all the equipment, I thought if I get infected with this virus and transmitted it to my family, if people would be hurt and die because of me’. (#7)
Going into the fire	‘…Not all COVID‐19 patients have severe clinical symptoms…there is no correlation between a patient's death and clinical symptoms…a patient with mild symptoms may die whereas another with severe symptoms recover…the unknown dimensions of the disease are numerous…’ (#1)
	‘…Uncertainty, …really uncertainty about everything…what will happen to the hospital, what will happen to us when we go home’. (#4)
	‘…going into the fire…because of the times of COVID and the severity of symptoms, you are going in with uncertainty’. (#5)
	‘COVID‐19 was a novel disease. I didn't know enough about it. We were frightened and scared. The number of confirmed cases and deaths was increasing greatly’. (#6)
Feeling guilty	‘In some ways it's a feeling of helplessness with these patients because you kind of sit there and watch them suffer and there's not a whole lot you can do about it’. (#2)
	‘Your training and learning are useless and you are just watching your patients slowly deteriorate and eventually die’. (#5)
	‘In the morning, the patient's breathing was still stable but, the next day, the patient could not breathe, the condition developed so rapidly. There were more and more confirmed patients, many of them could not be hospitalized in time, special vaccines have not yet been developed, so I was very stressed’. (#6)
	‘I was still feeding the patient water…after a while, the patient died…As a nurse, I feel guilty that I cannot save the patients’. (#10)
Burden of overloaded and unfamiliar work	‘In addition to routine work of monitoring of the patients, intravenous administration, and ventilator catheter care, the nurse also had to fully undertake life care such as changing the
	bed sheets, assisting with eating and excretion, cleaning the vomit’. (#3)
	‘Nurses I have never known or seen. They were assigned to our service unit from another one. I don't know their reactions…we had a dispute the other day with another Nurse…It feels as if working in another hospital. Different patients, a different order’. (#4)
	‘Most of them receive mechanical ventilation, some needs continuous renal replacement therapy or even extracorporeal membrane oxygenation. Mastering these skills quickly was a huge challenge for me’. (#6)
	‘The environment was unfamiliar and my colleagues were also unfamiliar. The operating procedures and disease care routines were different from previous work. I felt very anxious’. (#8)
Exhausted of wearing PPE (personal protective equipment)	‘…These clothes, these excessive precautions that we have to take because of our job, and the nature of the disease…we cannot take off or move our masks, glasses, or shields. It puts pressure on us…when your face itches, and you cannot touch it, this is very annoying…’ (#1)
	‘The body is airtight, breathing is a little difficult, the goggles still fog although we tried ways to avoid it. We all got soaked after a shift. All those situations seriously affected our performance’. (#3)
	‘Wearing protective clothing, the whole person will feel out of breath; 4–6 h without water intake, can't go to the toilet midway. After a day, the urine will soon become the color of sauce and oil, exhausted physically and mentally!’ (#6)
	‘When wearing the heavy protective equipment into the ward for the first time, I felt out of breath, chest tightness, and scared. The goggles were covered with fog in less than half an hour, and because of that, I could not see clearly’. (#9)

#### 5.2.1 Anxiety about infection and fear of transmitting the virus to their families

Nurses' most common psychological experiences during their first encounter with COVID‐19 were fear and anxiety. They experienced anxiety about becoming infected due to their work requiring close contact with patients (Galehdar et al., 2020; Gordon et al., 2021; Kackin et al., 2021; Lapum et al., 2021; Liu et al., 2020; Muz & Erdoğan Yüce, 2021; Sun et al., 2020; Zhang et al., 2020). Some developed compulsive thoughts and actions, such as thinking of every place as being contaminated and incessantly washing their hands and clothes to prevent infection (Galehdar et al., 2020; Kackin et al., 2021). Nurses feared being potential carriers of the virus and spreading it to family members and expressed hope that loved ones would not be infected (Galehdar et al., 2020; Gordon et al., 2021; Kackin et al., 2021; Lapum et al., 2021; Liu et al., 2020; Sun et al., 2020; Zhang et al., 2020). One nurse cried and stated that the infection risk to nurses and the risk of bringing the virus home to the family has led to very high stress (Gordon et al., 2021). Some nurses had minimal contact with their family members for several days because they feared transmitting the virus (Galehdar et al., 2020; Kackin et al., 2021; Lapum et al., 2021).

#### 5.2.2 Going into the fire

As COVID‐19 was a novel and unknown disease, front‐line nurses reported uncertainty due to the lack of information and experience (Galehdar et al., 2020; Gordon et al., 2021; He et al., 2021; Kackin et al., 2021; Lapum et al., 2021; Muz & Erdoğan Yüce, 2021; Sun et al., 2020; Zhang et al., 2020). During the early stage of COVID‐19, the unpredictable situation caused fear among nurses (Galehdar et al., 2020; Lapum et al., 2021; Liu et al., 2020; Muz & Erdoğan Yüce, 2021; Sun et al., 2020; Zhang et al., 2020). One nurse stated that working in this uncertainty is like going into a fire (Lapum et al., 2021). Early on, they experienced confusion as they were not prepared to protect themselves or provide patient care. They were unsure whether they could provide care safely and what care would be needed (Galehdar et al., 2020; Gordon et al., 2021; He et al., 2021; Lapum et al., 2021; Liu et al., 2020; Muz & Erdoğan Yüce, 2021; Sun et al., 2020; Zhang et al., 2020, 2021).

#### 5.2.3 Feeling guilty

Nurses reported psychological stress associated with the deterioration of patients' conditions. Despite nurses' efforts to help patients recover from COVID‐19, some patients' conditions worsened, and the nurses had to witness their deaths. Because they could do nothing, they felt helpless, powerless, sad and desperate (Galehdar et al., 2020; Gordon et al., 2021; He et al., 2021; Lapum et al., 2021; Liu et al., 2020; Muz & Erdoğan Yüce, 2021). One of the nurses described her experience of inevitably watching patients' conditions slowly deteriorate and eventually die (Lapum et al., 2021). They felt guilty about being unable to alter the situation; some nurses believed that they could not adequately support patients (He et al., 2021; Kackin et al., 2021; Lapum et al., 2021; Muz & Erdoğan Yüce, 2021; Zhang et al., 2021).

#### 5.2.4 Burden of overloaded and unfamiliar work

Excessive workload induced physical and psychological stress. Nurses had to care for critically ill COVID‐19 patients who required specialized and complex procedures. One nurse stated that most patients received mechanical ventilation, some needed continuous renal replacement therapy or extracorporeal membrane oxygenation, and it was a massive challenge for the nurse to quickly master these skills (Liu et al., 2020). The nurses said that it was a burden to quickly learn these skills while repeatedly putting on and removing PPE. They also suffered pressure to participate in disinfection, isolation and counselling patients and their families (He et al., 2021; Liu et al., 2020; Sun et al., 2020). In addition to high‐intensity work, working in an unfamiliar environment with new co‐workers caused stress and anxiety. Because new disease care routines and practices differed from their previous work, the nurses had to adapt to new working models and environments, resulting in emotional distress and confusion (Gordon et al., 2021; He et al., 2021; Kackin et al., 2021; Sun et al., 2020; Zhang et al., 2020, 2021).

#### 5.2.5 Exhausted of wearing PPE (personal protective equipment)

Wearing PPE posed physical and psychological challenges. Nurses used PPE, including N‐95 masks, goggles, gloves and gowns, every time they cared for COVID‐19 patients to prevent infection. Wearing PPE generated a great deal of pressure, heat, sweat and shortness of breath (Galehdar et al., 2020; Gordon et al., 2021; He et al., 2021; Lapum et al., 2021; Liu et al., 2020; Zhang et al., 2020). Wearing it caused physical discomfort and aggravated psychological exhaustion for nurses (Galehdar et al., 2020; Gordon et al., 2021; He et al., 2021; Lapum et al., 2021; Liu et al., 2020; Muz & Erdoğan Yüce, 2021; Sun et al., 2020; Zhang et al., 2020). They reported restrictions on drinking, eating and mobility while wearing PPE (Galehdar et al., 2020; He et al., 2021; Liu et al., 2020; Sun et al., 2020). Some nurses decided not to eat or drink until the end of their shifts because they had to take off their PPE, increasing their workload and wasting the PPE (He et al., 2021; Liu et al., 2020; Muz & Erdoğan Yüce, 2021). One nurse described that because she could not go to the bathroom during work, after work, the urine turned into the colour of ‘sauce’ or ‘oil’ (Liu et al., 2020).

### 5.3 Positive psychological experiences of nurses caring for COVID‐19 patients

Table 3 presents the positive psychological experiences of nurses caring for COVID‐19 patients, which are (1) calling of professional spirit, (2) a sense of accomplishment, (3) I am in a TEAM and (4) there is no resiliency without adversity.

**TABLE 3:** Positive psychological experiences of nurses in COVID‐19 (*N* = 10).

Themes	Quotations
Calling of professional spirit	‘In the face of the virus, feeling scared and wanting to escape from it is the instinct of people, but rushing to the front line is the calling of our professional spirit!’ (#3)
	‘Looked at nursing as part of a calling in my identity’ (#5)
	‘In this sudden outbreak, tens of thousands of nurses fought on the front‐line. I have been proud of nursing’. (#6)
	‘As a healthcare worker, I have the obligation and responsibility to treat patients who are suffering. As a nurse, my education prepares me with the ability to care and comfort patients and families’. (#9)
A sense of accomplishment	‘Many medical experts publicly praised nurses in the mass media; they praised the quality of nursing and talent, the attitude and team‐work of the nurses in the time of crisis’. (#6)
	‘During the pandemic, we proved to the society that the nursing profession is very important. At the moment, I think the society knows very well what we know, what training we have received, and our value’. (#7)
	‘Patients bowed to us when they were discharged. Their actions of appreciation touched me, which energized me and made me have a great sense of achievement’. (#9)
	‘I saw that the patient's breathing was stable, and the vital signs slowly returned to the normal range. I was really happy for them, and my heart felt inexplicably a sense of accomplishment’. (#10)
I am in a TEAM	‘I'm able to kind of share [my feelings] with my co‐workers and then…all my decompression happens’. (#2)
	‘…I feel like working on the battlefield, staffs of all ranks are united, and regardless of the title and grade, we are happy to work together. I am in a TEAM’. (#3)
	‘We thought about each other; this was very nice. Tried to protect each other. There was solidarity, a coming together as colleagues. The pandemic was a difficult, tough period. But I think we were closer to each other; I felt satisfied’. (#7)
	‘We encourage each other. It does not feel like I'm fighting alone, I'm not afraid’. (#8)
There is no resiliency without the adversity	‘there's no resiliency without the struggle, without the adversity’ (#5)
	‘The government has issued a series of effective policies, provided a large number of protective materials, and allocated a number of medical staff to the frontline. Many senior and experienced medical experts were also available to guide the work. I believed the epidemic would be overcome soon’. (#6)
	‘I never thought I could be so strong.”, “It feels like there's nothing I cannot overcome (laughs)’. (#8)

#### 5.3.1 Calling of professional Spirit

The nurses described it as their responsibility, duty, and mission to provide care to COVID‐19 patients despite the risk of infection (He et al., 2021; Lapum et al., 2021; Liu et al., 2020; Sun et al., 2020; Zhang et al., 2020, 2021). They stated that saving lives during the pandemic was inevitable and was their duty as a nurse (He et al., 2021; Liu et al., 2020; Sun et al., 2020; Zhang et al., 2020). One nurse revealed that rushing to the front line is the calling of their professional spirit (He et al., 2021). They also stated that their professional satisfaction and pride increased as they played a critical role in the global crisis (Lapum et al., 2021; Muz & Erdoğan Yüce, 2021; Zhang et al., 2021). During this difficult period, nursing tasks provided an opportunity to reevaluate the value of their roles and strengthened their professional identities (Lapum et al., 2021; Muz & Erdoğan Yüce, 2021; Sun et al., 2020; Zhang et al., 2020, 2021).

#### 5.3.2 A sense of accomplishment

When COVID‐19 patients praised the nurses and acknowledged their efforts, they felt great confidence and a sense of accomplishment (Liu et al., 2020; Muz & Erdoğan Yüce, 2021; Zhang et al., 2020, 2021). They were happy and proud when patients recovered and were discharged from the hospital. A nurse observed that when the patient's breathing stabilized and the vital signs slowly returned to the normal range, she felt satisfied and had an inexplicable sense of accomplishment (Zhang et al., 2021). Patients' affirmation and praise were positive incentives for front‐line nurses (Liu et al., 2020; Muz & Erdoğan Yüce, 2021; Zhang et al., 2020, 2021). Appreciation of the value of nursing from the media and society also bolstered their motivations (Muz & Erdoğan Yüce, 2021; Zhang et al., 2021). Additionally, the nurses expressed confidence that the pandemic would be overcome in the end. They believed that, with the help of citizens, governments, and international organizations, they would eventually win against the virus (Liu et al., 2020; Sun et al., 2020).

#### 5.3.3 I am in a TEAM

During the pandemic, nurses helped, supported and valued their colleagues with an altruistic attitude (He et al., 2021; Muz & Erdoğan Yüce, 2021; Sun et al., 2020). A nurse stated that staff of all ranks were united in a team regardless of the title and grade, and they were happy to work together (He et al., 2021). The pandemic caused a sense of professional solidarity, team cohesion and cooperation (He et al., 2021; Muz & Erdoğan Yüce, 2021; Sun et al., 2020). They shared similar feelings with their colleagues, both fellow nurses and other medical staff on their teams, and team support made them feel cared for and respected (Gordon et al., 2021; Sun et al., 2020; Zhang et al., 2020).

#### 5.3.4 There is no resiliency without the adversity

The nurses had the will to overcome the risks by adapting. They believed that they could overcome COVID‐19 and eventually succeed (Lapum et al., 2021; Liu et al., 2020; Sun et al., 2020). They also believed that they could learn from the challenging experience. They thought that such adversity would become a foundation to make nurses stronger, helping them recover from any difficult situations in the future (Lapum et al., 2021; Liu et al., 2020; Sun et al., 2020). One nurse expressed that she felt there was nothing she could not overcome (Sun et al., 2020). The belief that there is no resiliency without adversity strengthened their willpower, helped develop their potential and gave them the courage to face the struggles of life (Lapum et al., 2021; Sun et al., 2020).

### 5.4 Coping strategies of nurses caring for COVID‐19 patients

Table 4 presents the strategies nurses used to cope with their experiences of caring for COVID‐19 patients, which were (1) expressing emotions through crying, (2) positive thinking, (3) avoidance, (4) distraction and mind/body wellness, (5) patient and public praise, (6) social support and (7) rewards and workplace environment.

**TABLE 4:** Coping strategies of nurses in COVID‐19 (*N* = 10).

Themes	Quotations
Expressing emotions through crying	‘…I am not someone who cries a lot, but I am crying’. (#4)
	‘We held it together on the unit, but…you'd go home, you tear up at the slightest thing…I just release my stress through crying…I cry things out in the shower or when I'm in bed’. (#5)
	‘I cannot help crying when I'm under too much pressure and I feel relaxed after crying’. (#8)
Positive thinking	‘Let's say it is work ethics…I know this is the job I have to do…’ (#4)
	‘I've often thought how much better it would be if the epidemic disappeared when I wake up…’, ‘In fact, the chance of medical staff infection is very low and the protection of negative pressure wards is better than other departments’. ‘The medical staff in Wuhan is really tough and some have sacrificed their own life. Compared with them, we feel that we are lucky, so my mental state has improved’. (#8)
Avoidance	‘I do not watch any news in the evening, I follow them on the Internet. I muted all the WhatsApp groups, I check them out for about 5 mins when I am available…to see if there is anything involving me…I protect myself like this…”, “I tried not to think at first. I think more in the hospital. When I come home, I go to my room and try not to have close contact with family members’. (#4)
	‘don't even allow ourselves to have feelings, because how else would we cope and survive?’ (#5)
	‘My method is not to think about stress, I shield it out of my life’. (#8)
Distraction, mind/body wellness	‘Music helps me a lot. Uplifting music’. ‘That's what I do more often, do more cooking and cleaning’. ‘Going on walks with the dogs is nice, or exercising. I like to exercise’. ‘I have that hour to myself to meditate…that has helped me tremendously for sure’. ‘I work out a lot that is also a good stress reliever for me that kind of helps stress get off my brain so that's always nice’. (#2)
	‘…I've been cooking more, making up new recipes’ (#4)
	“I write a diary and sometimes write letters’. ‘When I rest, I watch comedy, listen to meditation music, or practice yoga’. ‘I feel sleep is the best stress relief, I just want to sleep’. ‘I think eating and drinking will increase my resistance’. ‘The progressive breathing relaxation method recommended by my colleague is good, I often use it’. (#8)
Patient and public praise	‘Many medical experts publicly praised nurses in the mass media; they praised the quality of nursing and talent, the attitude and team‐work of the nurses in the time of crisis’. (#6)
	‘Online reviews say we are heroes…’ (#8)
	‘Patients bowed to us when they were discharged. Their actions of appreciation touched me, which energized me and made me have a great sense of achievement’. (#9)
Social support	‘I've been talking on the phone a lot with my best friend from forever…”, “My family helps me get through stuff too’. (#2)
	‘…We do not know coping strategies…I feel like consulting an expert, so it would be much better if psychosocial support were to be provided by psychologists, therapists in related fields by making appointments…We really need some sort of support, because we are under a lot of risk’. (#4)
	‘I really appreciate the people who care for and support me and I cherish this emotion’. (#8)
	‘That was my first in‐depth conversation with our director, which is unforgettable and makes me feel warm’. (#9)
Reward and workplace environment	‘There are still not enough nurses. Because lack of staff who knows intensive care is felt too much. New appointments have been made, but they are also very recent graduates. The number of nurses is low’. (#4)
	‘Though facilities and human resources were much better than before, I think it is not enough. From the early stage of the epidemic to the establishment of the cabin hospital for centralized isolation, tens of thousands of confirmed patients were added during this period. We need a system to respond quickly in a similar situation’. (#6)
	‘We have seen that the nursing profession is in the background financially and morally. We really want to get satisfaction both financially and spiritually’ (#7)
	‘The hospital has an extra bonus for us and we have priority in promotion. The union also gave us gifts and expresses sympathy to us’. ‘A lot of companies donated money and supplies to support our fight against the epidemic, and I was very moved!’ ‘[institution] also actively paid for our antiepidemic health insurance; it feels like everyone is supporting us’. (#8)

#### 5.4.1 Expressing emotions through crying

Nurses relieved stress through the act of crying and felt more at ease after releasing their suppressed emotions (Kackin et al., 2021; Lapum et al., 2021; Sun et al., 2020; Zhang et al., 2021).

#### 5.4.2 Positive thinking

Some nurses assumed that the pandemic situation was temporary and that working in a negative pressure ward was safer than working in other departments. They accepted that they were fortunate to work outside of Wuhan, China, compared with medical staff in that city. They even imagined that COVID‐19 would disappear (Kackin et al., 2021; Sun et al., 2020).

#### 5.4.3 Avoidance

Some nurses tried to circumvent negative feelings through avoidance. They deliberately avoided thinking about or acknowledging stress and fear. They tried to protect themselves by limiting the use of media or news reporting negative information about COVID‐19 (Kackin et al., 2021; Lapum et al., 2021; Sun et al., 2020; Zhang et al., 2021).

#### 5.4.4 Distraction and mind or body wellness

The nurses used various distractions and mind or body wellness methods. They attempted to distract themselves by writing diaries, listening to music, watching comedy shows, eating, cooking and cleaning (Gordon et al., 2021; Kackin et al., 2021; Sun et al., 2020). In addition, they tried to relieve stress through sleep, yoga, meditation, breathing relaxation, mindfulness‐based stress reduction and exercise (Gordon et al., 2021; Sun et al., 2020).

#### 5.4.5 Patient and public praise

When a COVID‐19 patient was discharged from the hospital after recovering and heaped affirmation and praise on the nurses, the nurses felt touched and motivated. Praise from the media also became an opportunity to increase nurses' psychological strength and increase their pride (Liu et al., 2020; Sun et al., 2020; Zhang et al., 2020).

#### 5.4.6 Social support

Team support from colleagues was helpful in relieving stress. They shared their feelings and were motivated and encouraged by great teamwork (Gordon et al., 2021; He et al., 2021; Sun et al., 2020). Some nurses stated that support from the nursing manager, such as psychological comfort, shift adjustment and protection training, helped them relieve physical and psychological stress (Zhang et al., 2020). Support from family members and friends was also effective in enduring challenging situations (Gordon et al., 2021). Some nurses stated that psychosocial support from psychologists or therapists would be better for relieving their negative emotions (Kackin et al., 2021).

#### 5.4.7 Rewards and workplace environment

Some nurses received bonuses and received priority for promotions because of their care for COVID‐19 patients, which led to psychological and financial satisfaction (Sun et al., 2020). However, most nurses did not receive financial benefits and demanded increases in compensation. They stated that they should be rewarded for their work during the pandemic because they played an important role in managing the life‐threatening disease. They also stated that their rights in terms of nursing status and retirement should be improved (Muz & Erdoğan Yüce, 2021). They proposed that healthcare systems should be better prepared and respond more quickly to manage the crisis (Liu et al., 2020; Muz & Erdoğan Yüce, 2021). They suggested that increasing the number of nurses, adjusting work hours and shifts, initiating psychological interventions and providing protective equipment and supplies would be helpful for nurses' psychological well‐being and welfare (Kackin et al., 2021; Liu et al., 2020; Zhang et al., 2020).

## 6 DISCUSSION

This study reviewed 10 qualitative studies to synthesize the psychological experiences of nurses caring for COVID‐19 patients. While most previous studies have focused on the negative experiences of nurses fighting COVID‐19, this study presented the negative and positive experiences of front‐line nurses and their coping strategies. The empirical results of this study, which reviewed the psychological experiences of nurses during the early and acute phase of COVID‐19, published from December 2019 to September 2021, may contribute to developing and providing psychological support for nurses in the early stages of future pandemics.

The primary findings of this study indicate that nurses working during the pandemic experienced significant psychological concerns. Previous studies reported high levels of anxiety, fear, burnout and PTSD among nurses during severe acute respiratory syndrome (SARS) and the Middle East respiratory syndrome (MERS) outbreaks (Holroyd & McNaught, 2008; Kim, 2018; Maunder et al., 2006; Tam et al., 2004), aligning with this study's results. The fear of infection and transmitting the virus to family led to stress during the pandemic (Carmassi et al., 2020; Khalid et al., 2016; Shih et al., 2007). As front‐line nurses have direct contact with COVID‐19 patients, who require additional time for monitoring, titration, mobilization and hygiene compared to non‐COVID‐19 patients (Bruyneel et al., 2021), they experience increased psychological distress regarding the high risk of infection. This study also indicated that nurses experienced guilt and helplessness because of the aggravation and death of COVID‐19 patients. Patient deaths could negatively impact nurses' mental health. According to Mosheva et al. (2021), healthcare workers in COVID‐19 wards witnessed a higher proportion of patient deaths and experienced increased post‐traumatic stress symptoms than those in non‐COVID‐19 wards. Nurses witnessing the sudden deterioration or death of COVID‐19 patients may become traumatized (Levi & Moss, 2022). In addition, the nurses in this study experienced uncertainty about COVID‐19 due to a lack of knowledge and experience, leading to fear during the pandemic. This is consistent with the psychological experiences of nurses in previous epidemics such as SARS and MERS (Holroyd & McNaught, 2008; Lee et al., 2005, 2020). The speed of the outbreak, the unpreparedness of control measures and frequent changes in processes led to panic, fear and psychological distress (Holroyd & McNaught, 2008; Shih et al., 2007). Co‐workers' deaths from the infection increased uncertainty and fear of the disease (Holroyd & McNaught, 2008; Lee et al., 2005). Our findings also showed that wearing PPE was unpleasant and uncomfortable and led to psychological exhaustion. Other studies have reported that nurses wearing PPE during an infectious disease experience excessive sweating, dyspnea, fatigue and emotional exhaustion (Davey et al., 2021; Kim, 2018; Lee et al., 2005). According to Davey et al. (2021), wearing PPE during COVID‐19 caused healthcare workers to feel heat, stress and discomfort. The heat stress may reduce productivity, increase work‐related accidents (Flouris et al., 2018) and impair the quality of patient care (Lee et al., 2005). In addition, emotional distress due to unfamiliar work and the heavy workload was another stressor among nurses in this study, consistent with previous studies from outbreaks such as SARS, MERS and Ebola (Lee et al., 2020; Liu et al., 2019; Maunder et al., 2006). For all these reasons, it is essential to emphasize that nurses treating COVID‐19 patients often face negative experiences and should be assessed for their mental health. As COVID‐19 is an ongoing disease compared to previous SARS and MERS outbreaks, studies on PTSD relating to COVID‐19 are still lacking (Carmassi et al., 2020). Therefore, further studies should be conducted on the long‐term effects of PTSD symptoms in nurses facing COVID‐19 (Levi & Moss, 2022). For front‐line nurses, it is important to provide timely intervention strategies such as counselling, stress management and psychological interventions to improve their mental health. Providing nurses with the latest knowledge and scientific evidence and allowing them to participate in decision‐making would help reduce uncertainty and psychological distress during the pandemic (Levi & Moss, 2022; Shih et al., 2007). Additionally, nurse leaders should provide adequate and consistent guidelines regarding the procedures and protective measures for nurses caring for COVID‐19 patients. Establishing these guidelines may have a positive effect on patient safety and the psychological aspects related to nurses' work. Moreover, future studies on appropriate PPE‐use duration during a shift and a cooling environment are required for front‐line workers. Adjusting PPE designs to ensure safety and reduced heat strain is also required (Davey et al., 2021).

The nurses in this study perceived some positive aspects of the pandemic, despite the challenging circumstances. Caring for COVID‐19 patients was seen as a professional commitment and a responsibility. Nurses in this study reported that they renewed their appreciation of the essence of their profession. These results align with previous studies on epidemics such as SARS, influenza, and MERS, indicating that most healthcare workers were willing to continue working due to their professional obligations, despite the potential risk (Khalid et al., 2016; Lee et al., 2005; Seale et al., 2009). Tam et al. (2007) found that nurses developed a strong sense of identity and sought meaning in their profession from SARS experiences. Those who cared for SARS patients reframed their unique nursing experiences and became more robust and better prepared for future outbreaks. Nurses in this study also found increased professional pride, accomplishment, and confidence, aligning with Johal and Mounsey (2015), who found that nurses experienced increased confidence after providing care in a post‐earthquake and disaster‐stricken environment. These experiences gave them pride in their achievements and contributions to society. Additionally, when nurses received recognition for their contributions from patients or the media, pride in their profession was further strengthened in this study. Similarly, nurses who cared for MERS patients felt proud of providing quality care during a national disaster and felt rewarded when patients and other staff members praised nurses' efforts (Kim, 2018).

To cope with negative psychological experiences, nurses caring for COVID‐19 patients in this study used various strategies. They used adaptive strategies, such as expressing emotions, health‐promoting behaviours, and social support, and maladaptive strategies, including avoidance. These results are consistent with a previous study on SARS (Lee et al., 2005) that indicated some nurses dealt with their work positively by engaging in activities such as exercise, balanced diet, rest, internet surfing and connecting with families and friends to relieve tension. However, others avoided watching the news about SARS on television, which led to increased pressure and stress as they failed to accept reality. Previous studies indicated that positive coping strategies such as seeking information or social support and problem‐solving efforts alleviate psychological distress (Lou et al., 2022; Su et al., 2007; Yan et al., 2021), while damaging and avoidant strategies aggravate emotional distress, such as stress, burnout and PTSD, during an epidemic (Lou et al., 2022; Maunder et al., 2006). Therefore, interventions that actively engage nurses in collaborative planning for outbreaks and encourage sound working relationships with effective leadership may reduce maladaptive strategies and promote adaptive strategies (Lee et al., 2005; Maunder et al., 2006). Studies by Johal and Mounsey (2015) and Kim (2018) found that good relationships with colleagues were the driving forces enabling nurses to endure and overcome national disasters (Johal & Mounsey, 2015). It has also been reported that colleagues' positive attitudes helped ease healthcare workers' stress during MERS (Khalid et al., 2016). Effective teamwork, where nurses share information and support each other, can help them adapt to new conditions, reduce work burden and ease negative emotions.

In a pandemic situation, where other infectious diseases may occur after SARS, MERS and COVID‐19, consistent education and policy support should be provided for front‐line nurses to prepare for and cope with psychological issues. The guidelines of infection controls and procedures should be standardized and educated for nurses. Moreover, the latest information and scientific evidence should be informed to the nurses. Further research is needed on the long‐term psychological effect of nurses during the pandemic. In addition, there is a need for policy research on how healthcare institutions and governments can respond quickly to the pandemic and provide timely interventions to healthcare workers.

### 6.1 Implications for clinical nursing

This study highlighted psychological problems prevalent among nurses during COVID‐19. However, most were working without adequate supportive interventions. Strategies in nursing practice, research and policy are needed to support nurses fighting the pandemic on the front lines: (1) psychological support to improve front‐line nurses' mental health, especially regular and continuous psychiatric services; (2) sufficient rest and appropriate work shifts to reduce psychological stress and burnout; (3) support from family members and colleagues to relieve negative emotions; (4) financial support to recognize and encourage their hard work and (5) sufficient equipment and supplies to ensure safety. Beyond providing supportive interventions at the individual and organizational levels, we suggest providing adequate support resources for front‐line nurses globally. A network and collaboration of governmental health systems must be formed to help nurses communicate and utilize psychological counselling services and the latest knowledge about the disease in future health emergencies.

### 6.2 Limitations

This integrative review had several limitations. First, five of the 10 studies reviewed in this research were conducted in China, which should be carefully considered in generalizing the results worldwide. Second, as COVID‐19 information is changing rapidly and vaccines and treatments are being developed, nurses' feelings about COVID‐19 may change over time. Because most of the literature in this study dealt with nurses' psychological experiences when the situation peaked in the early stages of COVID‐19, its findings should be interpreted with caution. Third, since this review was limited to literature published in English or Korean, nurses' experiences in various countries where COVID‐19 was widespread were excluded.

## 7 CONCLUSION

This integrative review revealed that front‐line nurses during the COVID‐19 pandemic had positive and negative psychological experiences and used various strategies to cope with psychological distress. These results demonstrate the need for nursing practice, research and policy support for nurses to improve mental well‐being. It is vital for healthcare institutions and governments to provide support systems for nurses to ensure safe working environments and their psychological health.

## FUNDING INFORMATION

None.

## CONFLICT OF INTEREST STATEMENT

None.

## ETHICS STATEMENT

IRB submission was exempt due to this research being a review of data.

## Supporting information


Appendix S1.

## Data Availability

The data that support the findings of this study are openly available at http://doi.org/10.1002/nop2.1813.
