# Supplementary material for: Psychological experiences of nurses caring for patients with COVID‐19: Integrative review based on qualitative research
Source: Nurs Open. 2023 May 19;10(8):4919–31. doi: 10.1002/nop2.1813 (PMC10333851; doi:10.1002/nop2.1813)
Supplement: Supplementary file 1 — Appendix S1. [file NOP2-10-4919-s001.docx]

**Appendix 1**. Quality Appraisal of Selected Studies using the Critical Appraisal Skills Programme

| Sections | Section A | | | | | Section B | | | | Section C |
| --- | --- | --- | --- | --- | --- | --- | --- | --- | --- | --- |
| Broad issues | Are the results of the study valid? | | | | | What are the results? | | | | Will the results help locally? |
| Questions | Q1 | Q2 | Q3 | Q4 | Q5 | Q6 | Q7 | Q8 | Q9 | Q10 |
| Galehdar *et al.* (2020) | Yes | Yes | Yes | Yes | Yes | Yes | Yes | Yes | Yes | Yes |
| Liu *et al.* (2020) | Yes | Yes | Can’t tell | Yes | Yes | Yes | Yes | Yes | Yes | Yes |
| Muz *et al.* (2020) | Yes | Yes | Yes | Yes | Yes | Yes | Yes | Yes | Yes | Yes |
| Sun *et al*. (2020) | Yes | Yes | Yes | Yes | Yes | Yes | Yes | Yes | Yes | Yes |
| Zhang *et al*. (2020) | Yes | Yes | Yes | Yes | Yes | Yes | Yes | Yes | Yes | Yes |
| Gordon *et al*. (2021) | Yes | Yes | Yes | Yes | Yes | Yes | Yes | Yes | Yes | Yes |
| He *et al*. (2021) | Yes | Yes | Can’t tell | Yes | Yes | Yes | Yes | Yes | Yes | Yes |
| Kackin *et al*. (2021) | Yes | Yes | Can’t tell | Yes | Yes | Yes | Yes | Yes | Yes | Yes |
| Lapum *et al*. (2021) | Yes | Yes | Yes | Yes | Yes | Yes | Yes | Yes | Yes | Yes |
| Zhang *et al*. (2021) | Yes | Yes | Yes | Yes | Yes | Yes | Yes | Yes | Yes | Yes |

Note. Q1. Was there a clear statement of the aims of the research? Q2. Is a qualitative methodology appropriate? Q3. Was the research design appropriate to address the aims of the research? Q4. Was the recruitment strategy appropriate to the aims of the research? Q5. Was the data collected in a way that addressed the research issue? Q6. Has the relationship between researcher and participants been adequately considered? Q7. Have ethical issues been taken into consideration? Q8. Was the data analysis sufficiently rigorous? Q9. Is there a clear statement of findings? Q10. How valuable is the research?
